# Body image and related factors in patients with lower extremity lymphedema and lipedema: a cross-sectional study

**DOI:** 10.1590/1806-9282.20250274

**Published:** 2025-10-27

**Authors:** Sedef Ersoy, Fatma Nur Kesiktaş, Büşra Şirin Ahısha, Cansın Medin Ceylan, Nurdan Paker, Derya Buğdaycı

**Affiliations:** 1 Istanbul Physical Medicine and Rehabilitation Training and Research Hospital – Istanbul, Turkey.; 2 Beylikduzu State Hospital – Istanbul, Turkey.

**Keywords:** Body image, Lipedema, Lymphedema, Quality of life

## Abstract

**OBJECTIVE:**

Chronic edema in the lower extremities leads to significant negative effects on the quality of life, body image perception, satisfaction, self-confidence, and self-esteem of affected individuals. The aim of this study was to evaluate body image, quality of life, and related factors in patients with chronic lower extremity edema due to lymphedema and lipedema.

**METHODS:**

This cross-sectional study included 14 lymphedema and 12 lipedema patients receiving treatment at the lymphedema unit. Individuals aged 18–65 years with a confirmed diagnosis were enrolled; those with active infections, malignancies, or systemic diseases were excluded. Body image, dysfunctional thoughts about appearance, and quality of life were evaluated using the Body Cathexis Scale, Beliefs About Appearance Scale, and Lymphedema Quality of Life scale. Circumference measurements of the lower extremities were taken before and after 20 sessions of manual lymphatic drainage therapy. Quantitative data were analyzed to compare the two groups and assess correlations between clinical and psychosocial parameters.

**RESULTS:**

No significant differences were observed between the lymphedema and lipedema groups in terms of age, body mass index, or pre-treatment Body Cathexis Scale, Beliefs About Appearance Scale, and Lymphedema Quality of Life scale scores (p>0.05). After 20 sessions of manual lymphatic drainage therapy, both groups showed reductions in limb circumference measurements (p<0.05). Reductions in limb size were moderately associated with improvements in Body Cathexis Scale and Lymphedema Quality of Life scale scores (p<0.05). Post-treatment improvements in body image and quality of life scores were observed in both groups.

**CONCLUSIONS:**

This study highlights that patients with lower extremity lymphedema and lipedema experience significant body image disturbances and reduced quality of life. Manual lymphatic drainage therapy improves limb circumference, body image, and quality of life.

## INTRODUCTION

Lower extremity lymphedema is a chronic and difficult-to-treat condition caused by impaired lymphatic circulation, either congenitally or secondary to inflammation, malignancies, trauma, or iatrogenic causes^1^. Lipedema, on the other hand, is a chronic, progressive, and painful fat disorder characterized by disproportionate subcutaneous fat accumulation, typically affecting women. It is associated with pain, easy bruising, and tenderness upon palpation, and does not respond to weight loss^2^.

Despite differences in etiology and pathophysiology, both conditions lead to chronic lower extremity swelling, limb enlargement, heaviness, and pain, which in turn contribute to impaired physical function, body image disturbance, and reduced quality of life (QOL)^3–5^. Due to overlapping clinical manifestations and the use of similar conservative treatment strategies such as manual lymphatic drainage (MLD), these two conditions were evaluated together in the present study. MLD was applied as part of a comprehensive complex decongestive therapy (CDT) protocol, including skin care, therapeutic exercises, and compression therapy. While CDT is the gold standard for lymphedema management, it has also shown efficacy in alleviating symptoms in lipedema^3,4^.

While numerous studies have explored body image and QOL in patients with upper extremity lymphedema secondary to breast cancer, research on lymphedema and lipedema affecting the lower extremities remains scarce^6,7^. Therefore, this study aims to assess body image, QOL, and appearance-related thoughts in individuals with chronic lower extremity edema due to lymphedema and lipedema, and to explore the relationships among these factors.

## METHODS

This study enrolled 14 patients diagnosed with lymphedema and 12 patients diagnosed with lipedema, who received treatment at the lymphedema unit of Istanbul Physical Medicine and Rehabilitation Training and Research Hospital in May 2024. Ethical approval for this study was obtained from the Ethics Committee of Istanbul Physical Medicine and Rehabilitation Training and Research Hospital (Institutional Review Board approval number: 2024-22; approval date: April 30, 2024). Informed consent was obtained from all patients. This cross-sectional study was conducted and reported in accordance with the Strengthening the Reporting of Observational Studies in Epidemiology (STROBE) guidelines.

Demographic information including age, height, weight, and body mass index (BMI) of the patients was documented. Only patients with secondary lymphedema were included in the study. Lymphedema diagnosis was made clinically based on history (e.g., pelvic cancer surgery, radiotherapy, and recurrent infections) and physical examination findings including asymmetry, pitting edema, skin changes, and a positive Stemmer sign. The stage of lymphedema was determined according to the International Society of Lymphology (ISL) classification. In this staging system, Stage 0 refers to a latent phase where lymph transport is impaired but swelling is not yet evident. Stage I is characterized by soft pitting edema that improves with elevation. Stage II involves persistent swelling with increasing fibrosis that does not fully regress with elevation, and Stage III includes severe swelling with significant skin thickening, fibrosis, and trophic skin changes. Lipedema was diagnosed based on the 2020 European Lipoedema Consensus criteria, which include symmetrical fat accumulation predominantly in the lower extremities with sparing of the feet, pain or tenderness on palpation, a tendency to bruise easily, and resistance to weight loss^8^. The body image, dysfunctional thoughts about appearance, and QOL of the patients were evaluated using the Body Cathexis Scale (BCS), Beliefs About Appearance Scale (BAAS), and Lymphedema Quality of Life (LYMQOL) scale.

All participants underwent a total of 20 sessions of Phase I CDT, delivered 5 days per week over 4 consecutive weeks. Each session lasted approximately 60 min. CDT was administered in accordance with the 2023 consensus report of the ISL, ensuring standardization of therapeutic procedures across all patients. The CDT protocol included four main components: skin care and hygiene education; MLD using low-pressure, rhythmic strokes in the direction of lymphatic flow; multi-layer short-stretch compression bandaging; and therapeutic exercises focusing on activation of the muscle pump while bandaged. All treatments were performed by a physiotherapist with over 5 years of clinical experience in lymphedema management, certified in MLD and CDT by a nationally recognized training program. This ensured consistency and adherence to standardized techniques during all sessions.

The presence and severity of lymphedema and lipedema were determined by measuring the circumference of the lower extremities. The circumference of each metatarsophalangeal (MTP) joint and the thinnest point above the ankle malleolus were measured, and leg diameter measurements were taken at 10, 20, and 30 cm proximal to the lateral malleolus. Measurements were repeated after the 20 sessions of MLD therapy. To minimize measurement bias, all circumference measurements were performed by a physiotherapist who was not involved in treatment delivery and was blinded to the study hypothesis and evaluation timing.

### The Body Cathexis Scale

It was first developed by Secord and Jourard in 1953^9^. It was translated into Turkish by Hovardaoğlu^10^. The scale assesses individuals’ satisfaction with 40 different body parts or functions. The scale consists of 40 items, and patients are asked to rate each item on a scale from 1 to 5. The total scores range from 40 to 200, with higher scores reflecting greater satisfaction^10^.

### Beliefs About Appearance Scale

This 20-item scale was developed to assess the presence of dysfunctional thoughts about appearance^11^. Its Turkish validity and reliability were established by a study conducted by Göçet Tekin et al. Higher scores indicate a greater presence of dysfunctional thoughts^12^.

### Lymphedema Quality of Life scale

This scale was developed by Keeley et al. in 2010 to assess the QOL in lymphedema patients. The Turkish validation and reliability of this scale were established by Borman et al^13,14^. The scale includes 21 questions covering categories such as function, appearance, symptoms, and mood, with each question rated from 1 to 4. In this questionnaire, a higher score indicates poorer QOL^14^.

### Sample size calculation

The sample size was calculated using G*Power 3.1 (Universität Düsseldorf, Germany). A previous study reported that complex decongestive physiotherapy significantly improved QOL in lower extremity lymphedema patients, with scores on the LYMQOL scale decreasing from 121.50±77.02 to 59.25±40.80, corresponding to an effect size of 0.90. Assuming a similar effect size, a minimum of 26 patients was required to achieve 80% power at a 95% confidence level^15^.

### Statistical analysis

Statistical analyses were performed using IBM SPSS Statistics 21.0. Data normality was tested with the Shapiro-Wilk test. As the data were non-normally distributed, non-parametric tests were used. The Mann-Whitney U test compared continuous variables between groups, and Spearman's correlation assessed associations among body image, appearance-related beliefs, and QOL. A p<0.05 was considered statistically significant.

## RESULTS

The mean age of participants was 57.27±11.5 years, and all were female. No significant differences were found between the lymphedema and lipedema groups in terms of age, BMI, pre-treatment BCS, BAAS, LYMQOL scale scores, or circumference measurements (p>0.05). However, lipedema patients showed poorer psychological adjustment and greater impairments in physical and social functioning.

Significant negative correlations were found between BCS and LYMQOL scale scores (r=-0.574, p=0.002) and between BCS and BAAS scores (r=-0.572, p=0.002). No significant associations were found with age or BMI (Table 1). Both groups showed significant reductions in extremity circumference after 20 MLD sessions (p<0.05); however, the degree of improvement did not differ significantly between the groups (p>0.05) (Table 2).

**Table 1:** Correlation between body image and other parameters.

	Body Cathexis Scale	
	Rho	p
Lymphedema Quality of Life scale	**-0.574**	**0.002** p
Beliefs About Appearance Scale	**-0.223**	**0.002** p
Age	−0.210	0.303^p^
Body mass index	−0.315	0.117^p^

p Pearson's test. Statistically significant values are denoted in bold.

**Table 2:** Evaluation of the difference between pre- and post-treatment limb circumference measurements.

	Lymphedema (mean±SD)	Lipedema (mean±SD)	p-value	Effect size
MTP	−1.29±0.87	−1.21±1.56	0.376^m^	−0.174
Ankle	−2.68±3.00	−1.54±2.02	0.211^m^	−0.245
10 cm	−4.14±3.04	−2.96±2.87	0.196^m^	−0.253
20 cm	−3.00±1.94	−1.95±1.34	0.131^t^	−0.621
30 cm	−2.21±1.67	−2.63±3.01	0.816^m^	−0.046

m Mann-Whitney U test, tindependent sample t-test, MTP: metatarsophalangeal; SD: standard deviation.

Post-treatment changes in BCS scores were significantly correlated with reductions in all extremity circumference measurements (p<0.05). Additionally, improvements in QOL scores were associated with decreases at the ankle and 10 cm levels (p<0.05) (Table 3).

**Table 3:** Correlation between changes in Beliefs About Appearance Scale, Lymphedema Quality of Life scale, Body Cathexis Scale scores and limb circumference measurements after treatment.

	BAAS			LYMQOL scale			BCS		
	r	P	95%CI	r	p	95%CI	r	p	95%CI
30 cm	0.439*	0.025^s^	0.062–0.706	−0.261	0.197^s^	−0.589 to 0.141	−0.549**	0.004^s^	−0.772 to −0.205
20 cm	−0.082	0.689^p^	−0.455 to 0.315	−0.198	0.331^s^	−0.544 to 0.205	−0.548**	0.004^s^	−0.772 to −0.204
10 cm	−0.091	0.657^s^	−0.462 to 0.307	−0.444*	0.023^s^	−0.709 to −0.068	−0.602**	0.001^s^	−0.802 to −0.279
Ankle	0.071	0.729^s^	−0.325 to 0.446	−0.462*	0.018^s^	−0.720 to −0.091	−0.596**	0.001^s^	−0.799 to −0.271
MTP	0.092	0.654^s^	−0.306 to 0.463	−0.355	0.075^s^	−0.653 to 0.038	−0.509**	0.008^s^	−0.748 to −0.152

BAAS: Beliefs About Appearance Scale; LYMQOL: Lymphedema Quality of Life scale; BCS: Body Cathexis Scale; MTP: metatarsophalangeal; CI: confidence interval. Statistically significant values are denoted in bold.

* p<0.05,

** p<0.01.

s Spearman correlation test;

p Pearson correlation test.

To examine the potential influence of confounding variables on body image, a multiple linear regression analysis was conducted using the BCS score as the dependent variable. Independent variables included age, obesity (based on BMI classification), number of comorbidities, and disease stage. The results indicated that none of these variables had a statistically significant effect on body image scores (p>0.05 for all predictors). These findings suggest that body image perception in this patient group was not significantly influenced by these potential confounders within the limits of this sample.

## DISCUSSION

In this study, low body image scores were found in patients with chronic lower extremity edema due to lymphedema and lipedema. Additionally, poor QOL and dysfunctional thoughts about appearance were present. Following sessions of MLD, a reduction in lower extremity circumference measurements was observed in both groups, correlating with significant improvements in body image scores and QOL.

In the study by Teo et al., associations were found between pain levels, body image, and depressive symptoms in patients with upper extremity lymphedema related to breast cancer^6^. Another study highlighted prevalent concerns related to body image in patients with breast cancer-related upper extremity lymphedema^7^. Stolldorf et al.^1^ examined 213 patients with lower extremity lymphedema, showing concerns about appearance in 82.3% of patients, reduced physical activity in 70.3%, sadness in 68.6%, and loss of body confidence in 67.3%. A study conducted in Poland reported that patients with lipedema had reduced QOL and elevated depressive symptoms. It also demonstrated that worsening QOL was associated with symptom severity, pain, and swelling^16^. In the study by Yaman et al., patients diagnosed with lymphedema and lipedema were compared. While the rate of QOL impairment was similar between the two groups, life satisfaction was found to be lower in the lymphedema group^17^.

In the study by Cho et al., poor QOL in patients with lower extremity lymphedema secondary to gynecological cancer was found to be associated with lower satisfaction with body image^18^. Another study reported lower body image scores and body satisfaction in patients with upper extremity lymphedema, which was associated with decreased QOL^15^. In a study on melanoma-related limb lymphedema patients, lower QOL scores were observed^19^. In a study evaluating patients with lipedema, appearance-related concerns and depression were shown to significantly affect QOL^20^. In our study, consistent with the literature, a significant relationship was found between body satisfaction and QOL in patients with lymphedema and lipedema. Previous studies have also shown that lymphedema can negatively impact not only body image and psychosocial functioning but also sexual health and intimacy^6,7^. Similarly, chronic conditions such as polycystic ovary syndrome (PCOS), which can negatively affect self-image, have also been associated with altered sexual function and psychosocial distress. Although some studies did not find significant differences in sexual performance compared to healthy controls, concerns about appearance, menstrual irregularities, and emotional well-being were reported to influence sexual behavior in PCOS patients^21,22^. These findings collectively emphasize the broader relevance of body image issues and their psychosocial consequences in female-predominant chronic conditions involving visible physical changes.

In our study, both the lymphedema and lipedema groups showed significant reductions in extremity circumference and improvements in body image and QOL after 20 MLD sessions, although the degree of improvement did not differ significantly between groups. Similarly, Bongi et al. reported reduced limb volume and improved QOL following MLD in patients with upper extremity lymphedema secondary to systemic sclerosis^23^. Another study focusing on postmastectomy lymphedema demonstrated reductions in extremity swelling and improvements in QOL through rehabilitation in 51 patients^24^. Liu et al. applied 20 sessions of CDT to lower extremity lymphedema patients secondary to gynecological cancer, resulting in reductions in extremity circumference and improvements in QOL^25^. In the study by Atan et al., significant improvements in limb volume, pain, and functionality were observed in the group of 33 patients with lipedema who received CDT, and this group was found to be superior compared to the control group^26^. In their study, Szolnoky et al. demonstrated that CDT applied to patients with lipedema resulted in a significant reduction in lower extremity volume^27^. These studies in the literature support the findings observed in our study following treatment in patients with lymphedema and lipedema.

MLD therapy stimulates lymphatic circulation in patients with lymphedema through a pumping effect, facilitates the mobilization of edema, and assists in the removal of excess interstitial fluid. It has also been shown to reduce sympathetic response and inflammation^23^. In the literature, MLD therapy has also been reported to reduce pain and discomfort in patients with lipedema. It has been specifically shown to decrease sodium accumulation in the lower extremities, which is known to be associated with pain and inflammation^27,28^. Additionally, CDT has been shown to reduce capillary fragility in patients with lipedema, thereby decreasing the formation of hematomas^27^. Moreover, although lipedema is primarily a fat disorder, cases accompanied by lymphatic dysfunction are not uncommon. In such cases, where lymphatic dysfunction and skin folds are present, MLD may exert its effects through mechanisms similar to those observed in lymphedema^26^.

Our study has several strengths and limitations. Since most of the existing literature on body image and QOL focuses on upper extremity lymphedema following breast cancer, our study is one of the few that investigates these parameters in patients with lower extremity lymphedema and lipedema. All measurements were performed by the same specialist, ensuring procedural standardization, which we consider a methodological strength.

However, the small sample size increases the risk of both type I (false positive) and type II (false negative) errors. In particular, the possibility that statistically significant findings may be due to false positives cannot be ruled out, especially given the number of comparisons made in a relatively small sample. The single-center design and the lack of a control group further limit the generalizability of our findings and preclude the assessment of placebo effects or the isolated impact of MLD relative to other components of CDT. Moreover, only female patients were included in the study, which restricts the applicability of the results to male populations. Statistical analyses were based on pre-defined hypotheses, and no multiple comparison corrections were applied in order to preserve statistical power due to the small sample size. Nonetheless, the increased risk of type I error in the context of multiple comparisons should be taken into consideration when interpreting the results. Although validated Turkish versions of all assessment tools were used, cultural differences in body image and appearance perception may have influenced the responses. To examine the potential confounding effect of clinical parameters on body image outcomes, a regression analysis was conducted. However, mood-related factors such as depression and anxiety—which may significantly impact body image—were not assessed and thus not included in the analysis. This omission is acknowledged as a limitation. While pre- and post-treatment changes were evaluated, this study was observational in nature. Therefore, no causal inferences can be drawn. Future longitudinal or prospective randomized controlled studies are needed to better assess causality. Additionally, future research with larger sample sizes, multi-center participation, and more balanced gender representation will help generate more comprehensive and generalizable results.

## CONCLUSION

This study demonstrates that individuals with lower extremity lymphedema and lipedema experience substantial impairments in body image and QOL. In our cohort, CDT, including MLD, was associated with improvements in limb circumference, body image perception, and QOL scores. While these findings are encouraging, they should be interpreted with caution due to the limited sample size and lack of a control group. Future multicenter studies with larger sample sizes and more balanced gender distribution are needed to obtain more comprehensive and generalizable results.

## Data Availability

The datasets generated and/or analyzed during the current study are available from the corresponding author upon reasonable request.
